# Unraveling a mechanism underlying hepatitis E-associated kidney disease: Discovery of HEV ORF2 capsid protein-associated immune complex glomerulonephritis

**DOI:** 10.1007/s00292-025-01499-7

**Published:** 2025-11-24

**Authors:** Anne Laure Leblond

**Affiliations:** Institut für Pathologie und Molekularpathologie, PATH D57, Universitätsspital Zürich, Schmelzbergstraße 12, 8091 Zürich, Switzerland

**Keywords:** Liver pathology, Kidney pathology, Hepatitis E virus, Viral capsid protein, Immune complex-mediated glomerulonephritis, Leberpathologie, Nierenpathologie, Hepatitis-E-Virus, Viruskapsidprotein, Immunkomplexvermittelte Glomerulonephritis

## Abstract

**Background and objective:**

Hepatitis E virus (HEV) infection, one of the most common forms of hepatitis worldwide, is often associated with extrahepatic manifestations, particularly renal disease. While the underlying pathomechanisms are still largely unknown, these manifestations are thought to develop either directly, i.e., by HEV infection of the respective organ, or indirectly, i.e., via immunologic reactions. Herein, we describe the development of de novo immune complex-mediated glomerulonephritis (GN) associated with the glomerular deposition of a newly described form of the HEV open reading frame 2 (ORF2) capsid protein in patients with chronic or acute hepatitis E.

**Methods:**

We performed immunostaining, electron and deconvolution microscopy, and laser-capture microdissection combined with mass spectrometry to specifically investigate the glomerular compartment.

**Results:**

In a kidney transplant recipient with chronic hepatitis E, we show that GN developed in parallel with increasing glomerular deposits of the HEV ORF2 protein, which significantly colocalizes with IgG, thus forming immune complexes. Interestingly, the glomerular HEV ORF2 protein does not correspond to the expected secreted and glycosylated form of the viral capsid protein but rather has the molecular weight of a truncated non-glycosylated form. Importantly, it is not associated with HEV RNA and, in contrast to the situation in liver cells, no productive HEV infection of kidney cells is detected. Patients with acute hepatitis E show similar but less pronounced deposits. Our results establish a link between the production of HEV ORF2 protein and the development of hepatitis E-associated GN.

**Conclusion:**

The formation of glomerular IgG–HEV ORF2 immune complexes discovered here provides a mechanistic explanation of how the hepatotropic HEV can cause variable renal manifestations. These findings directly provide a tool for etiology-based diagnosis of hepatitis E-associated GN, establish hepatitis E-associated GN as a distinct entity, and suggest therapeutic implications.

## Abstract (de)

**Hintergrund und Ziel:**

Die Hepatitis-E-Virus(HEV)-Infektion, eine der häufigsten Formen der Hepatitis weltweit, ist oft mit extrahepatischen Manifestationen assoziiert, insbesondere mit Nierenerkrankungen. Während die zugrunde liegenden Pathomechanismen immer noch weitgehend unbekannt sind, wird davon ausgegangen, dass diese Manifestationen sich entweder direkt, d. h. durch eine HEV-Infektion des entsprechenden Organs, oder indirekt, d. h. über immunologische Reaktionen, entwickelt. In der vorliegenden Arbeit wird die Entstehung einer immunkomplexvermittelten De-novo-Glomerulonephritis (GN) dargestellt, die mit der glomerulären Ablagerung einer erstmals beschriebenen Form des HEV-Open-Reading-Frame-2(ORF2)-Kapsid-Proteins bei Patienten mit chronischer oder akuter Hepatitis E einhergeht.

**Methoden:**

Es erfolgten Immunfärbung, Elektronen- und Dekonvolutionsmikroskopie sowie Laser-Capture-Mikrodissektion im Kombination mit Massenspektrometrie, um insbesondere das glomeruläre Kompartiment zu untersuchen.

**Ergebnisse:**

Am Fall eines Nierentransplantatempfängers mit chronischer Hepatitis E wird gezeigt, dass die GN sich parallel mit zunehmenden glomerulären Ablagerungen des HEV-ORF2-Proteins entwickelte, das sich in signifikanter Weise mit Immunglobulin G (IgG) überlagert und so Immunkomplexe bildet. Jedoch entspricht das glomeruläre HEV-ORF2-Protein nicht den erwarteten sezernierten und glykosylierten Formen des Viruskapsidproteins, sondern weist eher das Molekulargewicht der trunkierten nichtglykosylierten Form auf. Wichtig ist, dass es nicht mit HEV-RNA assoziiert ist und, im Gegensatz zu der Situation in Leberzellen, keine produktive HEV-Infektion von Nierenzellen festgestellt wurde. Patienten mit akuter Hepatitis E weisen ähnliche, aber weniger ausgeprägte Ablagerungen auf. Durch die vorliegenden Ergebnisse wird ein Zusammenhang zwischen der Produktion von HEV-ORF2-Protein und der Entstehung einer Hepatitis-E-assoziierten GN hergestellt.

**Schlussfolgerung:**

Die hier erstbeschriebene Bildung von glomerulären IgG-HEV-ORF2-Immunkomplexen liefert eine mechanistische Erklärung dafür, wie die hepatotrope HEV variable Nierenmanifestationen verursachen kann. Mit diesen Ergebnissen besteht ein direktes Instrument für eine ätiologiebasierte Diagnose der Hepatitis-E-assoziierten GN, was die Hepatitis-E-assoziierte GN als eigene Entität etabliert und von therapeutischer Bedeutung sein kann.

The formation of immune-complex associated with HEV ORF2 capsid protein leads to the development of glomerulonephritis upon HEV infection.

## Background

Hepatitis E virus (HEV) infection is the leading cause of acute viral hepatitis in humans, contributing to an estimated 3.3 million symptomatic cases and nearly 70,000 deaths each year [1]. The WHO also indicates that one third of the world’s population is at risk of HEV infection, which is recognized as the main cause of viral hepatitis worldwide. In European countries, HEV infection frequently bears a high risk of developing into chronic hepatitis in immunocompromised individuals, particularly organ transplant patients [2, 3]. Thus, HEV imposes a global health burden in both resource-rich and resource-poor countries. Additionally, the virus can be transmitted vertically during pregnancy, via blood derivatives, via transplants, and through direct person–person contact [4, 5], which further complicates the transmission and the risk of HEV infection.

Most HEV infections are asymptomatic or subclinical and cause acute self-limiting hepatitis. However, the virus can also cause fulminant hepatitis, particularly in pregnant women, with a mortality rate of 20–30% in the third trimester. Acute HEV infections in individuals with pre-existing liver damage, such as cirrhosis, can lead to rapid liver decompensation, liver failure, and death [4]. Hepatitis E virus infection can also progress to a chronic or persistent state in immunocompromised individuals. Long-term manifestations can escalate from inflammation to liver fibrosis, cirrhosis, and death due to decompensation [6]. More recent findings, including those from our group, suggest that in addition to any pre-existing liver disease, the immune status of the host is a decisive determinant of the pathogenesis and course of HEV infection [7–9]. There is no specific treatment for acute-on-chronic liver failure due to HEV. Current therapeutic options are limited to off-label use of the nucleoside analogue ribavirin and pegylated interferon-alpha, if reducing immunosuppression does not eliminate the virus [10].

Although hepatitis E virus (HEV) primarily replicates in the liver, extrahepatic manifestations, especially renal injury, have frequently been reported [11, 12]. Most HEV-associated renal manifestations occur in immunocompromised individuals and are often overlooked in the clinical management of non-immunocompromised patients with acute hepatitis E, where attention is focused on liver function. Our understanding of HEV-associated renal injury and the different prognoses for the liver and kidney following HEV infection remains limited. It is conceivable that manifestations are due either to viral replication in infected tissues (as recently suggested [13, 14]) directly leading to cellular damage or that immunological reactions involving the formation of immune complexes indirectly cause cellular damage.

Central to the understanding of HEV pathogenesis is the genetic organization and lifecycle of the hepatitis E virus. Hepatitis E virus is a hepatotropic single-stranded, positive-sense RNA virus that encodes three main open reading frames (ORFs): ORF1 encodes non-structural proteins required for virus replication, ORF2 is translated into the viral capsid protein, and ORF3 encodes a small protein needed for the egress of the virions from infected cells [15]. As described in human serum and in vitro cell models, HEV produces different ORF2 isoforms with distinct molecular weights: a non-glycosylated intracellular isoform (ORF2intra) assembled into infectious particles (ORF2i) and glycosylated isoforms (ORF2g/c) secreted in large amounts and exhibiting thee sites of glycosylation at positions 137, 310, and 562 [16–18]. In addition to its three isoforms, HEV ORF2 protein has been found to interact with several cellular proteins and to play a role in apoptosis signaling in both model cell systems [19] and in vivo [20]. Research on vaccination against hepatitis E is based on this highly immunogenic protein and elicits virus-neutralizing antibody production [21]. Furthermore, the capsid protein is an important target of both the antiviral CD4 T‑cell [22] and the antibody response [23]. Thus, the multifaceted HEV ORF2 capsid protein illustrates how an RNA virus dysregulates many pathways in host cells, especially those involved in the immune response [24], to ensure complete and efficient replication of its genetic material [25].

## Objective

In the present study carried out in HEV-infected patients with impaired immune status, we investigated the implication of HEV ORF2 capsid protein, starting from the replication of the virus in the liver and the secretion of HEV ORF2 protein, to the formation of immune complexes in the glomeruli of the kidney. To this end, we performed careful histopathological and molecular analysis using powerful state-of-the-art microscopic and molecular biological analysis techniques.

## Results

**Fig. 1:**
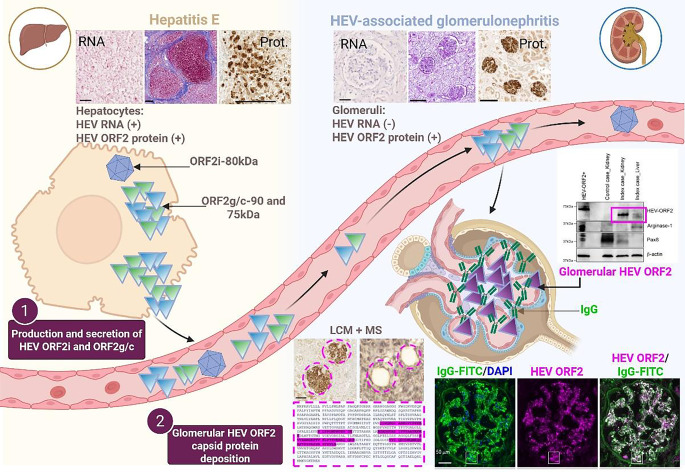
Graphical summary of the main findings published in Leblond A.L. and Helmchen B. et al., [26] demonstrating the causality between hepatitis E virus (HEV) infection and kidney failure in immunocompromised individuals due to the formation of HEV ORF2 protein/IgG immune complexes in renal glomeruli. *Prot.* protein, *LCM* laser capture microscopy and *MS* mass spectrometry. Created in BioRender.com

The results are summarized in Fig. 1 and published in [26].

In an immunosuppressed kidney transplant patient, strong immunoreactivity against the HEV ORF2 capsid protein [27] was detected not only in the liver but also in the kidney, more precisely in the glomeruli. Retrospective examination of kidney biopsies taken since the time of transplantation revealed that alongside the exacerbation of glomerular lesions and deterioration of kidney function, there was an increasing deposition of HEV ORF2 capsid in the glomeruli. Closer examination of the deposits was immediately suggestive of glomerulonephritis caused by immune complexes. Deconvolution microscopy confirmed this, showing a statistically significant co-localization of the HEV ORF2 capsid protein and immunoglobulin G.

Knowing that the HEV ORF2 capsid exists in different forms, we conducted a more thorough study to determine which form of the HEV ORF2 protein corresponds to the glomerular entity. To this end, we laser-captured the positively stained glomeruli and analyzed the protein content by mass spectrometry. This approach revealed that HEV ORF2 protein fragments were only detected in HEV-infected patients and almost exclusively in the glomeruli compared to the surrounding interstitial areas. Western blot experiments, both with and without endoglycosidase treatment, showed that this glomerular form corresponds to a truncated, non-glycosylated form. Using various methods, including in situ hybridization staining with an HEV RNA probe, PCR to determine viral load, and detection of HEV ORF1 and ORF3 using mass spectrometry, we were unable to detect infected kidney cells. This led us to conclude that there is no viral replication and that the glomerular HEV ORF2 protein is a non-infectious, genome-free form. In summary, we discovered that a previously undescribed, non-infectious, non-glycosylated form of the HEV ORF2 capsid protein accumulates in the glomeruli, forming immune complexes and triggering the development of glomerulonephritis.

## Implications for basic research and histopathologic diagnostics

With the description of the HEV ORF2 protein-mediated immunological mechanism of kidney damage, we have not only contributed to the fundamental understanding of the development of extrahepatic manifestations in the context of hepatitis E: our discovery also has direct implications for the histopathological diagnosis of hepatitis E-associated glomerulonephritis, as a proportion of immune complex glomerulonephritis can now be assigned to a specific etiology and reliably diagnosed using HEV ORF2 protein immunohistochemistry. We are confident that our discovery will improve the diagnosis of hepatitis E, particularly with regard to renal involvement. We hope that it will raise awareness of hepatitis E and help to ensure that this disease is not overlooked in the future. Those affected should benefit from this.

## Declarations

Written informed consent (provided by Springer Nature) was given by the next of kin of all patients according to CARE guidelines and in compliance with the principles of the Declaration of Helsinki. Clinical data have been anonymized. Sex and gender were not taken into consideration. This study was approved by the internal review board of the University Hospital Zurich and the Cantonal Ethics Committee of Zurich, Switzerland (KEK-ZH-Nr. 2013-0504).

The supplement containing this article is not sponsored by industry.
